# Historical fish survey datasets from productive aquatic ecosystems in Lithuania

**DOI:** 10.1016/j.dib.2022.107990

**Published:** 2022-02-23

**Authors:** Eglė Jakubavičiūtė, Freddie Heather, Giedrė Višinskienė, Augustas Morkvėnas, Harry Gorfine, Žilvinas Pūtys, Linas Ložys, Asta Audzijonyte

**Affiliations:** a Nature Research Centre, Akademijos g. 2, Vilnius, Lithuania; b Institute for Marine and Antarctic Sciences, University of Tasmania, Hobart, Tasmania 7005, Australia

**Keywords:** Fish surveys, Data standardization, Catch per unit effort, Fish size, Data sharing, Curonian lagoon, Kaunas water reservoir, Gillnetting

## Abstract

Many inland ecosystems (lakes, rivers, reservoirs, lagoons) around the world undergo regular biological monitoring surveys, including monitoring the abundance, biomass and size structure of fish communities. Yet, the majority of fish monitoring datasets for inland ecosystems remain inaccessible. This is especially true for historical datasets from the early and middle 20^th^ century, despite their immense importance for establishing baselines of ecosystem status (e.g., prior to manifestations of climate change and intensive fisheries impacts), assessing the current status of fish stocks, and more generally determining temporal changes in fish populations. Here we present a newly digitized fish monitoring dataset for two major Lithuanian inland ecosystems – Curonian Lagoon and Kaunas Water Reservoir. The data comprises >60000 records from >800 fish surveys conducted during 1950s to 1980s, using a range of fishing gears and sampling methods. We introduce three different definitions for survey methods to describe the level of detail for each fish community study. Method 1 surveys include individual fish sizes and weights, Method 2 surveys record frequencies of fish in length or weight groups, whereas Method 3 only records the total catch biomass of a given species. The majority of historical and currently collected fish survey data can be attributed to one of these three methods and we present R codes to convert data from higher resolution methods into aggregated data formats, to facilitate data sharing. In addition, commercial fisheries catch data for years that were surveyed are also provided. The data presented here can facilitate ecological and fisheries analyses of baseline ecosystem status before the onsets of rapid warming and eutrophication, exploration of fish size structure, evaluation of different catch per unit effort standardization methods, and assessment of population responses to commercial fishing.

## Specifications Table

**Table:** 

Subject	Ecology
Specific subject area	Fisheries ecology Studies of exploited organisms in marine and freshwater systems
Type of data	Table
How data were acquired	Fish abundance, biomass and sizes were surveyed by Nature Research Centre (Lithuania) using a range of gillnets and trawls during 1950–1980s. For each survey fish abundances, sizes and biomasses were recorded and stored in archival journals, currently available at the Nature Research Centre (Lithuania). In this dataset, the archival journals were digitized and data supplemented with all available information on gear types and fishing effort. Details of catches for each fishing survey were provided using three alternative survey methods (Method 1–3).
Data format	Raw
Description of data collection	In this study we digitized all available hard copy records of fish surveys conducted in Kaunas Water Reservoir and Curonian Lagoon, where at least some gear information was available. Gear information, such as mesh size and gear length or type, was described in as much detail as possible, but when specific details were not provided in the original archival journals, expert opinion was used to make a best guess and recorded in separate columns, defining broad categories (e.g., of the mesh size, gear length). The fish surveys were conducted using a range of fishing gears, including nets, trawls and traps, beach seine, and a range of different mesh sizes, gear lengths, and different times of the day. In many cases, selective gillnet series ranging from 18 to 80 mm (knot to knot) mesh size were used. The gillnets were typically set between 17:00 and 19:00 h and lifted the next day between 07:00 and 09:00 h, although different soak times have been used during the 40-year sampling period. Net lengths differed at different sampling periods. During the 1950–1960s a lot of surveys were done using a trawl net, for which catchability and surveyed area differ from gillnets. All available gear details are listed in the relevant columns, described below. For each fishing survey, details of recorded fish also varied. In some surveys only the total biomass per species was recorded, in others there were details of length frequencies of each species, or individual length and weight measurements. Sometimes different levels of detail were used for different fish within the same survey, which in the database are identified by Methods 1–3 (see Experimental Design, Materials and Methods section below)
Data source location	Institution: Nature Research Centre City: Vilnius Country: Lithuania Latitude and longitude for collected samples/data: Curonian Lagoon – 55.0236° N, 20.8904° E Kaunas Water Reservoir - 54.8604° N, 24.1394° E
Data accessibility	In a public repository: Repository name: Dryad Direct URL to data (currently for private viewing, until the publication of this manuscript): https://datadryad.org/stash/share/utksFXHnn3U9wiwbc3QPTMeOeKIq9OJU7fuPkovHE8Y doi:10.5061/dryad.612jm644p

## Value of the Data

• Freshwater and coastal ecosystems are highly vulnerable to global warming, pollution, intensive fishing, and other human impacts. Detailed fish monitoring data from the mid-20^th^ century, as presented here, is essential to establish baselines of ecosystem status and to assess human impacts in terms of the extent of change in ecological parameters and indicators.
• The data are useful for ecological and fisheries research, including studies of size structure in freshwater ecosystems, recreational and commercial fishing impacts, warming impacts on fish biomass, size composition, and relative abundance.
• The data presented here could also be used for exploring various approaches to catch-per-unit-effort standardization, assessments of stock responses to fishing, determining species interactions and changes in relative biomasses, as well as measuring interactions between body size and abundance for different species.

## 1 Data Description

The dataset comprises information about fish sampled during scientific surveys. Specifically, data columns include:

• Year, day, and month when the sampling was performed
• Water body (Curonian Lagoon or Kaunas Water Reservoir), and location where the survey sampling took place. If known, precise coordinates of the sampling location are provided.
• Fish species (common English and Latin names),
• Total length (in cm),
• Standard length (in cm)
• Total weight (in g)
• Sex (1 – male, 2 – female, 3 - juvenile)
• Gonad weight (in g)
• Gonad stage (from immature to spawning, I to VI)
• Age (as inferred from scales)
• Fat weight (in g)
• Gut fullness. Three-digit number reflects gut fullness at the beginning of the gut (first number), in the middle (second) and at the end (third number). 0 – empty, 5 – full. Sometimes only a general indication with one- or two-digit numbers were given.
• ‘exclude_from_weight_est’ column indicates that a specific record (usually for Method 2, where number of fish i.e., frequency in sequential size groupings is given) should not be used for estimating total weight of the catch for that specific day and location, because the total catch weight is indicated separately, where all the individually measured fish were weighed in bulk.
• Number of fish
• Catch weight (in g), available when fish were not measured individually
• Catch_length_min and catch_length_max – size bins (in cm) of the fish caught, when fish were not measured individually. This refers to Method 3 surveys.
• Gear type (gillnet, trawl, beach seine, fyke-net), total gear length (in m).
• Total_gear_length_broad defines general length categories of the gear used because specific lengths were not always available. “Very short”: <=20 m, “short”: 20-60 m, “medium”: 61-120 m, “long”: 121-300 m, “very long”: >300 m.
• Mesh size of the gear (in mm) indicates the mesh size of the gear used. In some cases, a range of mesh sizes were used and fish catches were recorded for all meshes combined. In this case mesh_size_min and mesh_size_max indicate ranges of mesh sizes. Mesh_size_broad (in mm) – based on expert judgement, rough categories of the mesh sizes are defined, when exact values were missing: “small” <=38 mm, “big” >38 mm, “full” – full range, from small to big mesh sizes, e.g., 17-70 mm.
• Soak time (in hours). Soak_time_broad – soak time categories defined based on expert opinion, when exact values (in h) were not available: “short” <=5 h, “medium” 6-12 h, “long” =>12 h.
• Depth (in m) – depth of the sampling location.
• Method (method1, method2, method3) – indicates whether (which) individual fish data category is available (see Materials and Methods section), enabling easy filtering for future analysis.

## 2 Experimental Design, Materials and Methods

**Fig. 1:**
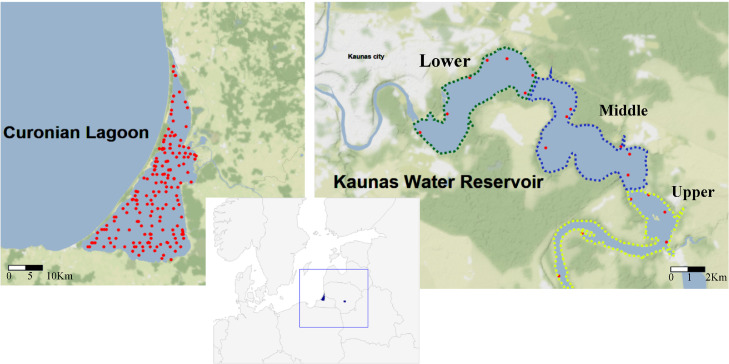
Map of the locations for which the fish monitoring datasets are provided – Curonian Lagoon, where red dots show sampling areas for which coordinates are known. Kaunas Water Reservoir, where dotted lines show division into three sections and sampling locations for which coordinates are known.

**Fig. 2:**
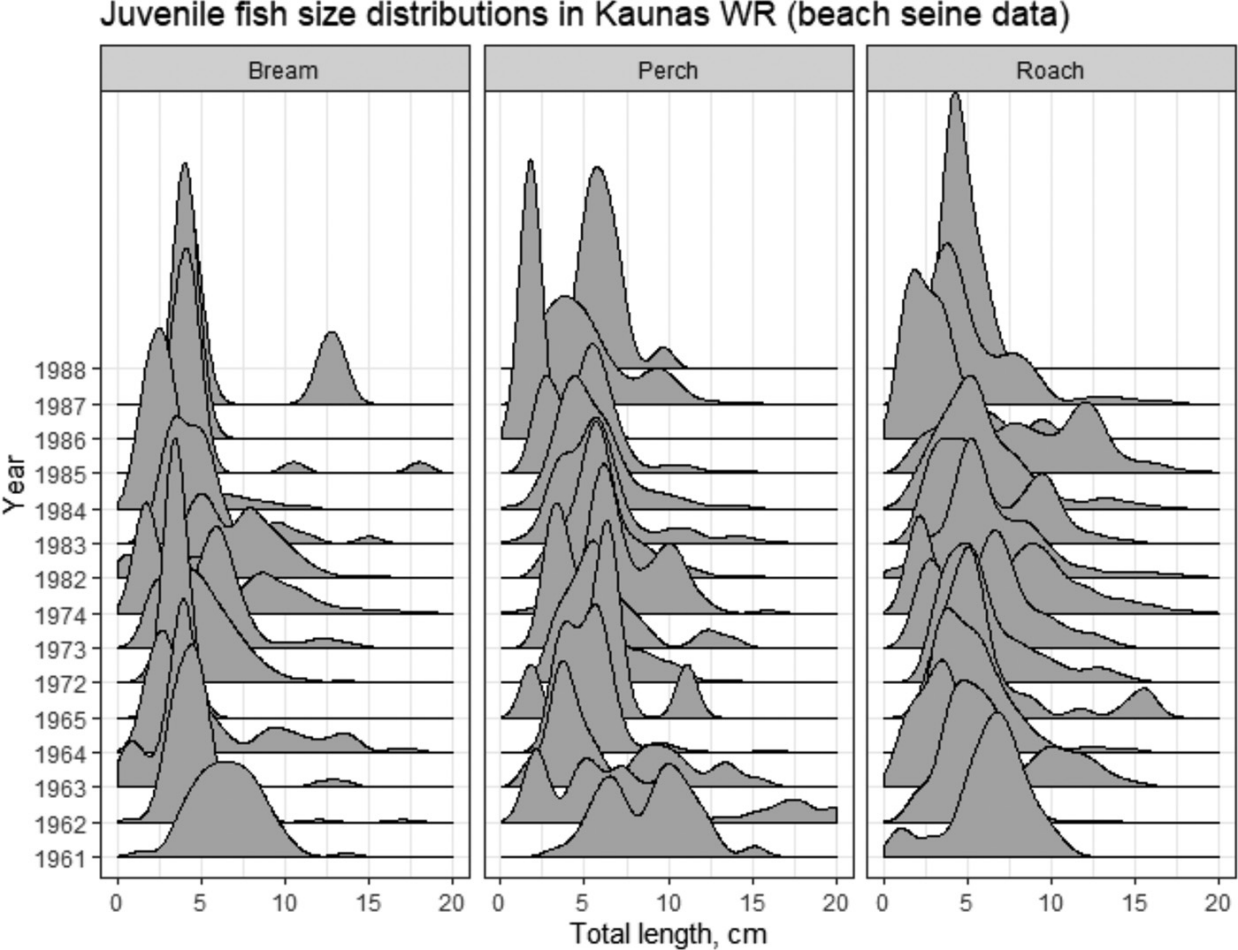
Size distributions of roach (N=5271), perch (N=4221) and bream (N=2131) from the juvenile fish community in Kaunas Water Reservoir, as collected by beach seine.

**Fig. 3:**
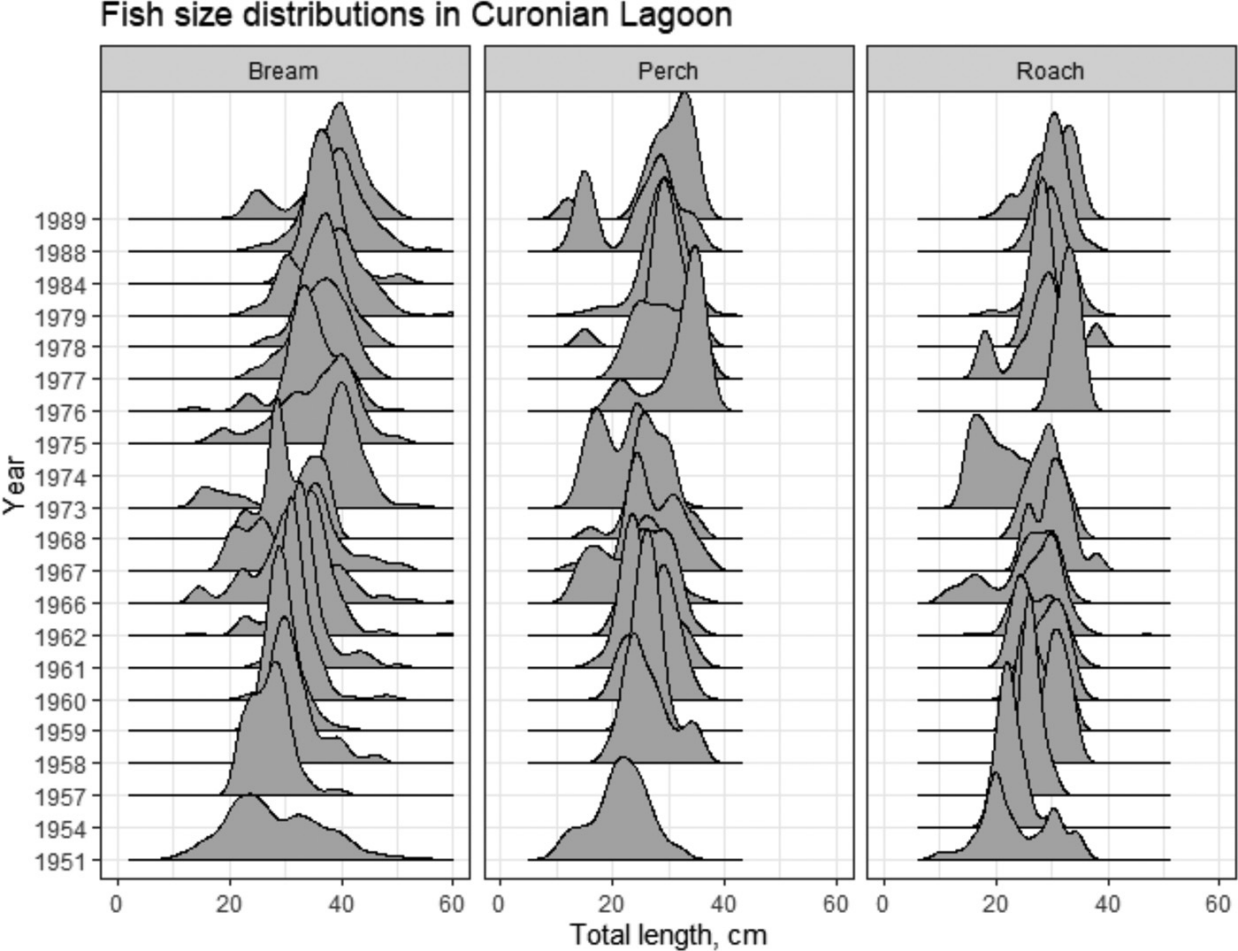
Size distributions of subadult and adult roach (N= 1739), perch (N= 1643) and bream (N= 11115) in the Curonian Lagoon over four decades, as collected by all gears except for beach seine.

**Fig. 4:**
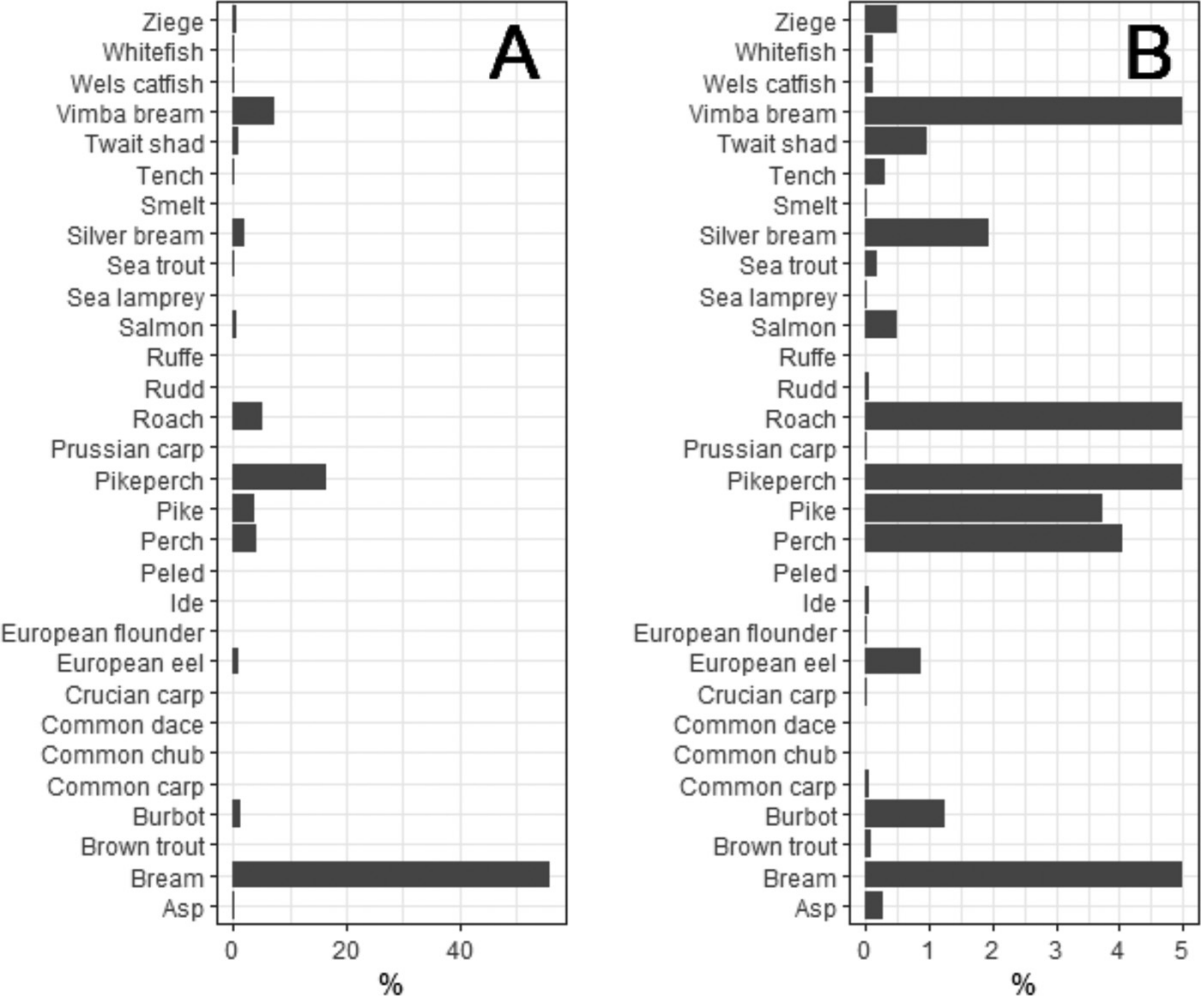
Fish species composition by biomass in the Curonian Lagoon. Panel B zooms into the lowest 5% to better show the relative biomass of uncommon species (for common species proportions were trimmed at 5%).

**Fig. 5:**
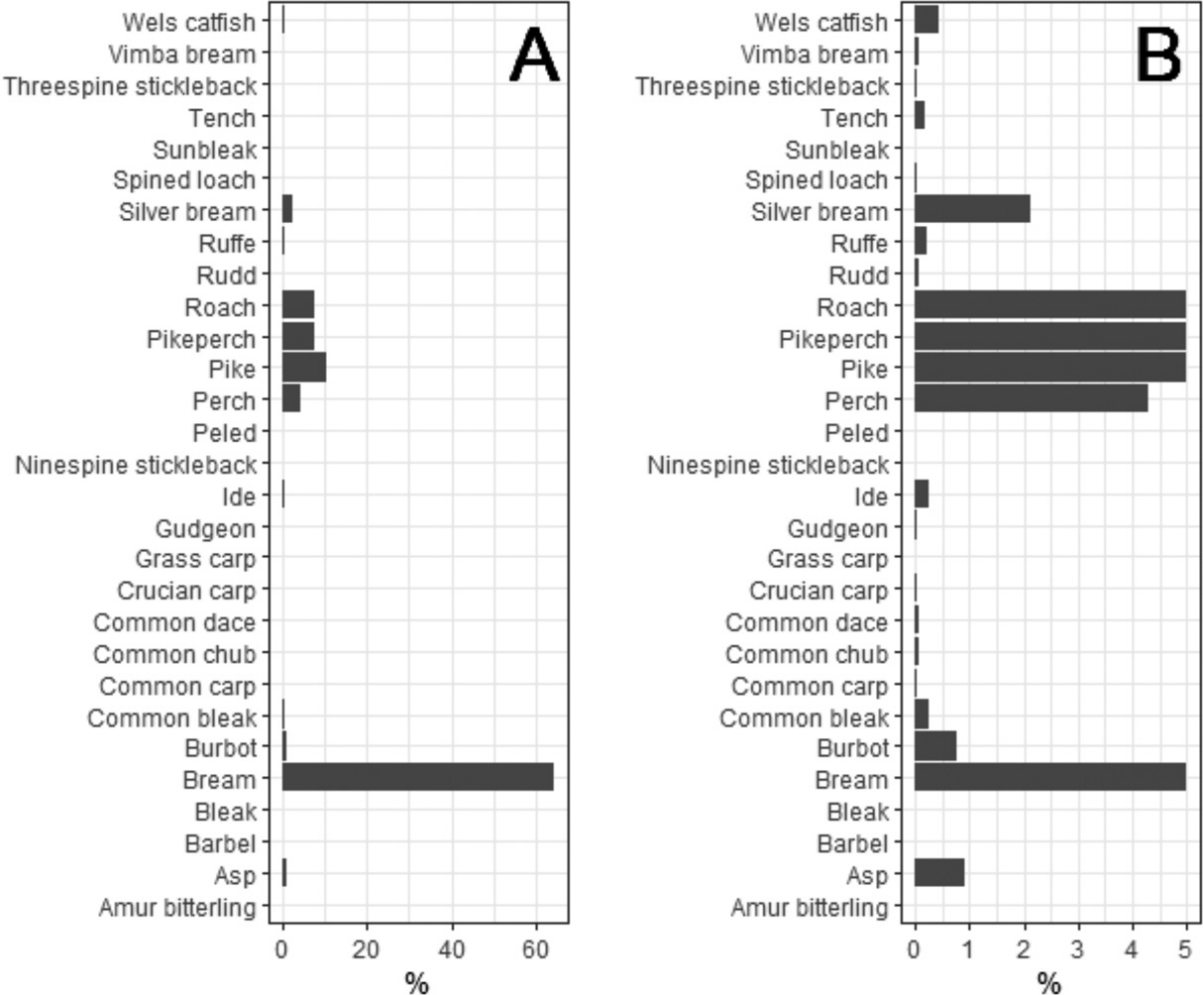
Fish species composition by biomass in Kaunas Water Reservoir. Panel B zooms into the lowest 5 % to better show relative biomass of uncommon species.

Approximately 40 years’ monitoring of fish communities has been undertaken at >150 locations in Curonian Lagoon (1951–1990) and for 30 years at 29 locations in Kaunas Water Reservoir (1961–1990), grouped into three main areas – Upper, Middle and Lower Reservoir (see Fig. 1) Fig. 2, Fig. 3, Fig. 4, Fig. 5. illustrate the types of data that have been collected during the four decades of surveys. Surveys were mainly performed using traditional gears: nets and trawls. A methodological shift from using predominately trawl to gill nets occurred in the 80s (mostly in Curonian Lagoon).

Fish caught were often measured and weighed individually. In some cases, only frequencies in fish length bins (in cm) and/or weight of total catch (in g) of a given species were recorded. To distinguish between these different types of data and to make it easy for filtering and future analyses, in each data row we assign the method of measurement:

• Method1 applies to surveys where fish were individually measured (total length and/or standard length) and weighed.
• Method2 indicates that fish were individually measured, often within 1 cm precision only, but not weighed.
• Method3 – only total catch weight is available, sometimes also the minimum and maximum lengths of the weighed catch are provided. For this method, length bins are larger than 1cm.

## Ethics Statement

Not applicable, because data acquisition does not involve working with humans or animal experiments.

## Supplementary Information

**Supplementary material 1.** Historical commercial fish catches in Curonian Lagoon and Kaunas Water Reservoir.

**Supplementary material 2**. R code to convert data from Method 1 (most detailed), into Method 2 and Method 3.

## CRediT Author Statement

**Eglė Jakubavičiūtė:** Data curation, Conceptualization, Methodology, Writing – original draft, Visualization; **Freddie Heather:** Software; **Giedrė Višinskienė:** Data curation; **Augustas Morkvėnas:** Data curation; **Harry Gorfine:** Methodology, Writing - review & editing; **Žilvinas Pūtys:** Methodology; **Linas Ložys:** Methodology, Data curation, Resources; **Asta Audzijonyte:** Supervision, Conceptualization, Methodology, Writing - review & editing, Funding acquisition.

## Declaration of Competing Interest

The authors declare that they have no known competing financial interests or personal relationships which have or could be perceived to have influenced the work reported in this article.

## Data Availability

Historical fish survey datasets from productive aquatic ecosystems in Lithuania (Original data) (Dryad).

## Appendix Supplementary Materials

Image, application 1

Image, application 2

Image, application 3

